# Extended-Spectrum Beta-Lactamase-Producing *Enterobacteriaceae* in Fresh Produce

**DOI:** 10.3390/foods10112609

**Published:** 2021-10-28

**Authors:** Alberto Pintor-Cora, Laura Álvaro-Llorente, Andrés Otero, Jose M. Rodríguez-Calleja, Jesús A. Santos

**Affiliations:** Department of Food Hygiene and Food Technology, Veterinary Faculty, Universidad de León, 24071 León, Spain; apintc@unileon.es (A.P.-C.); lalvl@unileon.es (L.Á.-L.); aotec@unileon.es (A.O.); jm.rcalleja@unileon.es (J.M.R.-C.)

**Keywords:** vegetables, ESBL-producing *Enterobacteriaceae*, *Serratia fonticola*, *Rahnella aquatilis*

## Abstract

Fresh vegetables are an essential part of a healthy diet, but microbial contamination of fruits and vegetables is a serious concern to human health, not only for the presence of foodborne pathogens but because they can be a vehicle for the transmission of antibiotic-resistant bacteria. This work aimed to investigate the importance of fresh produce in the transmission of extended-spectrum β-lactamases (ESBL)-producing *Enterobacteriaceae*. A total of 174 samples of vegetables (117) and farm environment (57) were analysed to determine enterobacterial contamination and presence of ESBL-producing *Enterobacteriaceae*. Enterobacterial counts above the detection limit were found in 82.9% vegetable samples and 36.8% environmental samples. The average count was 4.2 log cfu/g or mL, with a maximum value of 6.2 log cfu/g in a parsley sample. Leafy vegetables showed statistically significant higher mean counts than other vegetables. A total of 15 ESBL-producing isolates were obtained from vegetables (14) and water (1) samples and were identified as *Serratia fonticola* (11) and *Rahnella aquatilis* (4). Five isolates of *S. fonticola* were considered multi-drug resistant. Even though their implication in human infections is rare, they can become an environmental reservoir of antibiotic-resistance genes that can be further disseminated along the food chain.

## 1. Introduction

Consumption of fresh produce has risen worldwide as consumers grow interested in their nutritional values and their association with a healthy diet; thus the World Health Organization advises a daily intake of at least 400 g of fruit and vegetables. At the same time, microbial load of fruits and vegetables is a serious concern to human health, since a large portion of foods of plant origin are consumed raw, with a growing number of foodborne outbreaks linked to fresh produce [1,2,3]. Besides contributing to the spread of foodborne pathogens, an additional health concern is that vegetables represent a vehicle for the transfer of antibiotic-resistant bacteria or antimicrobial resistance genes to humans, which may occur through the consumption of contaminated fresh produce [4,5]. Recently, the European Food Safety Authority assessed the importance of several food-producing environments in the EU, including plant-based food production, in the emergence and spread of antimicrobial resistance [6]. This Scientific Opinion points to faecal microbial contamination of fertilisers, water and the production environment as specific interventions to minimise bacteria, such as carbapenem-resistant or extended-spectrum cephalosporin-resistant *Enterobacteriaceae*. Moreover, the report recognises the multiple data gaps as evidencing the importance of transmission routes leading to contamination with antibiotic-resistant bacteria at primary production and post-harvest stages of food-producing systems [6].

Extended-spectrum β-lactamases (ESBL) are enzymes conferring resistance to a great number of β-lactam antibiotics and there is an increased prevalence of members of the family Enterobacteriaceae producing ESBL, not only nosocomial, but also in the community [7,8]. The presence of ESBL-producing bacteria in vegetables has been reported in several studies [9,10,11,12,13,14] though the results are diverse, from the total absence of ESBL-producing bacteria [9] to isolation rates of 15% [10].

The order Enterobacterales, in which the family *Enterobacteriaceae* is included, has undergone a recent major revision [15], with the description of six additional new families. Throughout this work, the references to species of the family *Enterobacteriaceae* follow the former taxonomic outline defined in the last edition Bergey’s Manual [16].

Therefore, the aims of this work were to determine the importance of raw vegetables for direct human consumption as vehicles of extended-spectrum beta-lactamase-producing *Enterobacteriaceae* and their potential as environmental sources of contamination.

## 2. Materials and Methods

### 2.1. Sample Collection and Processing

A total of 117 samples of fresh vegetables were collected from two conventional farms, one organic farm and from a street market. All the vegetables were from Spain. Samples were of lettuce (*Lactuca sativa*, *n* = 23), tomato (*Solanum lycopersicum*, *n* = 19), cucumber (*Cucumis sativus*, *n* = 19), carrot (*Daucus carota* subsp. *sativus*, *n* = 18), escarole (*Cichorium endivia* var. latifolium, *n* = 13), pepper (*Capsicum annuum*, *n* = 10), parsley (*Petroselinum crispum*, *n* = 9) and coriander (*Coriandrum sativum*, *n* = 6). Fifty-seven additional samples from soil (*n* = 18), water (*n* = 14) and air (*n* = 13), as well as from the hands of farm workers (*n* = 12), were also taken in the farms.

Vegetables were lightly cleaned to remove foreign matter and nonedible parts were cut using sterile tools. Ten grams of each vegetable and soil sample were homogenised with 90 mL of buffered peptone water (BPW; Oxoid, Thermofisher, UK). Water samples were processed by filtering 100 mL of the sample through a 0.45 µm filter and soaking the filter in 100 mL of BPW. A volume of 100 L air-sample was collected in farms using a microbial air sampler (Biotest Hycon, Dreieich, Germany) fitted with a ChromAgar Enterobacteria plate (ChromAgar, Paris, France). One hand swab sample was taken from each farm worker, which was then soaked in a 10 mL of BPW tube.

BPW homogenates were diluted 1:10 in 0.1% peptone (Oxoid) and appropriate ten-fold dilutions were spread onto ChromAgar Enterobacteria plates (ChromAgar) and incubated at 37 °C for 24 h. Pink/reddish colonies were recorded as suspected *Enterobacteriaceae* and blue ones as suspected *E. coli*.

The remaining homogenates were incubated for 24 h at 37 °C. One loopful of the enriched solution was streaked onto ChromAgar ESBL (ChromAgar) plates for the isolation of ESBL-producing bacteria. Plates were incubated at 37 °C for 24 h and colonies with morphology associated with β-lactam resistance were selected for further characterisation.

### 2.2. Matrix-Assisted Laser Desorption Ionisation Time-of-Flight (MALDI-TOF) Identification of Isolates

The isolates were grown on Tryptone Soya Agar (TSA) plates (Oxoid) for 16–24 h at 37 °C. Colony material was collected with a sterile pipette tip and smeared as a thin film on a MALDI target plate. After air drying, each sample was overlaid with 1 µL of the matrix solution (α-Cyano-4-hydroxycinnamic acid, CHCA) and allowed to dry. Spectra were acquired with the MALDI Biotyper system (Bruker Daltonik, Bremer, Germany) and compared with the reference database (Bruker Daltonik).

### 2.3. Antimicrobial Susceptibility Testing

MAST D72C AmpC and ESBL detection kit (MAST group, UK) was used for ESBL confirmation. Equivocal results were confirmed using the combination disk diffusion test following the indications of EUCAST (https://eucast.org/; accessed on 29 September 2021). In addition, representative isolates were selected to determine minimum inhibitory concentration (MIC) by microdilution using Sensititre EUVSEC2 plates (Thermofisher, UK) with the following antimicrobials: cefoxitin, cefotaxime, cefotaxime/clavulanic acid, ceftazidime, ceftazidime/clavulanic acid, cefepime, ertapenem, imipenem, meropenem and temocillin. MIC interpretation was carried out according to EUCAST cutoff values.

*E. coli* CECT 434 was used as a negative control, while *E. coli* M1L9c was used as positive control for ESBL production.

The detection of multidrug-resistant (MDR) isolates was carried out using a selection of antimicrobial agents of different categories, according to the proposal of the European Centre for Disease Prevention and Control and the Centers for Disease Control and Prevention [17]. The antimicrobial agents were ampicillin, cefuroxime, cefotaxime, cefepime, aztreonam, imipenem, gentamicin, ciprofloxacin, trimethoprim-sulphamethoxazole and chloramphenicol [17].

### 2.4. PCR Detection and Characterisation of ESBL-Associated Genes

The presence of *bla_TEM_*, *bla_SHV_* and *bla_CTX-M_* genes in phenotypically confirmed ESBL-producing isolates was checked by PCR using the primers and conditions described elsewhere [18] (Supplementary Table S1).

Amplified products were purified and both strands were sequenced in a Mega-BACE 500 sequencer (GE Healthcare Life Sciences, UK). DNA sequences were compared with curated sequences contained in the Bacterial Antimicrobial Resistance Reference Gene Database (https://www.ncbi.nlm.nih.gov/bioproject/PRJNA313047; accessed on 29 September 2021).

### 2.5. Data Analysis

*Enterobacteriaceae* and *E. coli* counts were transformed and expressed as log cfu/mL or log cfu/g. Descriptive statistics of each count (mean, standard deviation, minimum and maximum) were calculated and, for each value of the agricultural groups (sample type, sample group, origin, and agricultural practices), normality/no normality assumptions about bacterial population distributions were examined. The Kruskal–Wallis one-factor ANOVA and the Mann–Whitney U procedure were used for testing the mean difference in counts among the groups and to determine their relationships. Subsequently, the nonparametric Kruskal–Wallis test was conducted for post-hoc pairwise comparisons on all possible pairwise contrasts. The IBM SPSS Statistics for Windows, Version 26.0 (IBM Corp., Armonk, NY, USA) program was used for data analysis.

## 3. Results

### 3.1. Enterobacteriaceae and E. coli Counts

Enterobacterial counts above the detection limit of 2 log cfu/g for vegetables and soil, and 1 cfu for sample volume for air, water and hands of workers were found in 97 (82.9%) vegetable samples and 21 (36.8%) environmental samples. The average count was 4.2 log cfu/g or mL, with a maximum value of 6.2 log cfu/g in a parsley sample. Leafy vegetables (lettuce, escarole, parsley and coriander) showed statistically significant (*p* < 0.05) higher mean counts than other vegetables (Table 1).

Twenty-nine (16.7%) samples presented counts of *E. coli* above the limit of detection, all but one obtained from vegetables, with an average value of 2.3 log cfu/g; five of them showed values higher than 3 log cfu/g, the upper limit established in the microbiological process hygiene criteria for precut ready-to-eat fruits and vegetables in the EU (Table 1) [19].

### 3.2. Isolation of ESBL Strains

Suspected ESBL isolates were obtained from Chromagar ESBL plates in 31 (17.8%) samples. Most of the suspected samples (26) were of vegetables, 20 of leaf vegetables and 6 of other vegetables. Five environmental samples of water (3), soil (1) and hands of workers (1) carried presumptive isolates. Confirmation with MAST D72C kit resulted in 15 ESBL-producing isolates, 14 from vegetables and 1 from a water sample (Table 2).

The detection of ESBL-producing bacteria was statistically related (*p* < 0.05) to the concentration of *Enterobacteriaceae* found in the sample, with a significant effect detected in the samples with *Enterobacteriaceae* counts above 5 log cfu/g.

The isolates were identified as *Serratia fonticola* (11) and *Rahnella aquatilis* (4).

Genes encoding CTX-M β-lactamases were detected in six (40%) isolates, whereas no ESBL-genes were detected with the specified primers in the remaining nine isolates. The *ctx-M* gene was detected in four isolates of *S. fonticola* and two of *R. aquatilis*, all of them isolated from vegetables. Analysis of sequences revealed that the genes from *S. fonticola* were similar to *bla_FONA5_* genes and those from *R. aquatilis* were similar to *bla_RAHN2_* gene (Table 2).

Five isolates of *S. fonticola* were resistant to antimicrobial agents of different categories, thus being considered MDR. No isolate was resistant to imipenem, ciprofloxacin, trimethoprim-sulphamethoxazole or chloramphenicol (Table 2).

Five *S. fonticola* and four *R. aquatilis* isolates representative of the positive samples were selected to determine minimum inhibitory concentrations (MIC). All the isolates showed an intermediate MIC value for temocillin. Three *R. aquatilis* and one *S. fonticola* isolate had MICs above the clinical breakpoint for cefotaxime and a reduction greater than two-fold concentration in the presence of clavulanic acid, which could be explained by the presence of ESBL enzymes. The remaining isolates had a detectable MIC value for cefotaxime below the breakpoint, again showing a reduction greater than two-fold in the presence of clavulanic acid. One *R. aquatilis* and two *S. fonticola* isolates had MICs above the breakpoint for cefoxitin, and one *S. fonticola* isolate for cefepime as well (Table 3).

## 4. Discussion

*Enterobacteriaceae* and *E. coli* counts are used as indicators of faecal contamination and lack of hygiene during food production, but there is a growing concern as many strains are becoming resistant to antibiotics used to treat human infections, such as carbapenems or colistin [20]. The results obtained in this study show that ready-to-eat fresh produce, especially leaf vegetables, presented high counts of *Enterobacteriaceae* and, to a lesser extent, of *E. coli*. The results are in accordance with similar works, thus Falomir et al. [21] detected coliforms counts in 50% of vegetable samples, carrot and lettuce being the most contaminated, suggesting the probability of contact with a source of contamination (soil, water, manure) during growth [14,22]. In another study, Mukherjee et al. [23] reported an overall prevalence of *E. coli* of 8% positive samples, being higher in leaf vegetables but lower than the prevalence found by us (16.7%), and an average count of 3.1 log MPN/g (2.3 cfu/g in our study). The environmental sample showing *E. coli* counts was taken from the hands of a farm worker, highlighting the importance of good food hygiene in reducing the microbial load of ready-to-eat foods [24,25].

The isolation of ESBL-producing *Enterobacteriaceae* in vegetable samples in this study confirmed previous reports that pointed to vegetables as a vehicle of dissemination of resistance genes [10,11,12,13,14]. The detection of ESBL-producing bacteria was related to *Enterobacteriaceae* counts, indicating that routine monitoring of this bacterial group in fresh produce is a good indicator both of hygiene quality and presence of antibiotic-resistant bacteria.

The isolates were identified as *Serratia fonticola* and *Rahnella aquatilis*. Antibiotic-resistant *S. fonticola* and *R. aquatilis* have been found in vegetables and farm environments [10,12,26,27,28,29] and their presence is considered of minor importance from a health point of view, as they are linked to human diseases in rare instances [30,31]. However, they can be the source of resistant genes which can be transferred to other bacteria causing human infections [32]. In addition, the ingestion of nonpathogenic ESBL-producing bacteria may result in potential intestinal horizontal gene transfer to pathogens [33]. It is important to keep in mind that the plasmid-encoded SFO-1 ESBL produced by *Enterobacer cloacae* is derived from the FONA enzyme of *S. fonticola* and has been observed in hospital outbreaks of infection in Spain [34,35]. Furthermore, the detection of MDR among *S. fonticola* isolates is an additional concern as other reports detected no MDR *S. fonticola* in vegetable samples [27] and a review of clinical cases of *S. fonticola* infections reported susceptibility to cefepime and gentamicin among the strains tested [36], whereas resistance to both antimicrobials was found in this study, indicating a recent acquisition of resistance to those agents. A recent report isolated a multidrug-resistant strain from a patient with a biliary tract infection [37], highlighting the relevance of dissemination of resistance genes among opportunistic bacteria.

As *S. fonticola* and *R. aquatilis* are considered environmental *Enterobacteriaceae*, irrigation water and soil are the probable route of contamination of vegetables, as suggested by other authors [10,14,38].

In conclusion, ESBL-producing *S. fonticola* and *R. aquatilis* are environmental *Enterobacteriaceae* commonly found in vegetables and other food commodities. Even though their implication in human infections is rare, they can become an environmental reservoir of antibiotic-resistant genes that can be further disseminated along the food chain.

## Figures and Tables

**Table 1:** Counts (log cfu/g or mL) of *Enterobacteriaceae* and *E. coli* in vegetables and environmental samples.

		*n*	*Enterobacteriaceae* Counts ^1^	*E. coli* Counts ^1^
	Lettuce	23	4.1 ± 1.1 (19)	2.7 ± 1.6 (3)
	Escarole	13	5.4 ± 0.3 (13)	3.0 ± 0.0 (1)
	Parsley	9	5.3 ± 0.5 (9)	2.4 + 1.0 (5)
	Coriander	6	5.1 ± 0.8 (6)	1.7 ± 0.0 (4)
Leaf vegetables		51	5.0 ± 1.0 (47)	2.5 ± 0.9 (13)
	Tomato	19	3.6 ± 0.8 (12)	2.3 ± 0.9 (4)
	Cucumber	19	3.9 ± 0.8 (11)	1.7 ± 0.0 (3)
	Pepper	10	3.5 ± 0.5 (10)	2.7 ± 0.9 (4)
	Carrot	18	4.7 ± 0.9 (17)	1.8 ± 0.2 (4)
Other vegetables		66	3.9 ± 0.9 (50)	2.1 ± 0.8 (15)
Total vegetables		117	4.5 ± 1.0 (97)	2.3 ± 0.8 (28)
	Soil	18	3.3 ± 0.8 (13)	- ^2^
	Water	14	3.0 ± 0.4 (6)	-
	Air	13	0.6 ± 0.4 (4)	-
	Worker hands	12	3.7 ± 0.6 (2)	2.8 ± 0.0 (1)
Environmental		57	2.9 ± 1.2 (21)	2.8 ± 0.0 (1)
Total		174	4.1 ± 1.2 (118)	2.3 ± 0.8 (29)

^1^ Average count ± standard deviation in log cfu/g or mL. In brackets, number of samples showing counts above the detection limit. ^2^ Counts below the detection limit (2 log CFU/g for vegetables and soil, and 1 cfu for sample volume for air, water and hands of workers).

**Table 2:** ESBL-producing *Enterobacteriaceae* isolated from vegetables and farm environment samples.

Isolate	ID	Source	Antibiotic Resistance Profiles ^1^	MDR	ESBL Gene
ZA07E1	*Rahnella aquatilis*	Carrot	AMP, CXM, CTX	No	*bla_RAHN2_*
CI03E	*Serratia fonticola*	Coriander	AMP, CXM	No	*bla_FONA5_*
CI10E	*Serratia fonticola*	Coriander	AMP, CXM, CTX, CN	Yes	*bla_FONA5_*
CI04E1	*Serratia fonticola*	Coriander	AMP, CXM, CTX	No	
PE11E	*Serratia fonticola*	Cucumber	AMP, CXM, CN	Yes	
ES09E	*Serratia fonticola*	Escarole	AMP, CXM	No	
ES48E	*Rahnella aquatilis*	Escarole	AMP, CXM, CTX	No	*bla_RAHN2_*
ES16E	*Rahnella aquatilis*	Escarole	AMP, CXM, CTX	No	
LE18E	*Serratia fonticola*	Lettuce	AMP, CXM, CTX	No	*bla_FONA5_*
PJ02E	*Serratia fonticola*	Parsley	AMP, CXM, CTX, FEP, ATM	Yes	
PJ07E	*Serratia fonticola*	Parsley	AMP, CXM, CTX, ATM	Yes	
PJ21E	*Serratia fonticola*	Parsley	AMP, CXM, CTX	No	
PJ27E	*Serratia fonticola*	Parsley	AMP, CXM, CTX, CN	Yes	*bla_FONA5_*
TO30E	*Rahnella aquatilis*	Tomato	AMP, CXM, CTX	No	
AG24E	*Serratia fonticola*	Water	AMP, CXM	No	

^1^ AMP, Ampicillin; CXM, Cefuroxime; CTX, cefotaxime; FEP, cefepime; ATM, aztreonam; CN, gentamicin.

**Table 3:** Minimum inhibitory concentrations of five *S. fonticola* and four *R. aquatilis* isolates.

Isolate	ID	Source	FOX (0.5–64)	FOT (0.25–64)	FC (0.06/4–64/4)	TAZ (0.25–128)	TC (0.12/4–128/4)	FEP (0.06–32)	ETP (0.015–2)	IMI (0.12–16)	MERO (0.03–16)	TRM (0.5–128)
ZA07E1	*Rahnella aquatillis*	Carrot	2	4 *	<0.06/4	<0.25	<0.12/4	0.25	<0.015	<0.012	<0.03	8 **
CI03E	*Serratia fonticola*	Coriander	4	0.5	<0.06/4	<0.25	<0.12/4	<0.06	<0.015	<0.012	<0.03	4 **
CI04E1	*Serratia fonticola*	Coriander	8 *	1	<0.06/4	<0.25	<0.12/4	<0.06	<0.015	<0.012	<0.03	8 **
ES16E	*Rahnella aquatillis*	Escarole	1	2 *	<0.06/4	<0.25	<0.12/4	0.12	<0.015	<0.012	<0.03	4 **
ES48E	*Rahnella aquatillis*	Escarole	8 *	2 *	<0.06/4	<0.25	<0.12/4	0.12	<0.015	<0.012	<0.03	4 **
LE18E	*Serratia fonticola*	Lettuce	2	0.5	<0.06/4	<0.25	<0.12/4	<0.06	<0.015	<0.012	<0.03	2 **
PJ02E	*Serratia fonticola*	Parsley	16 *	>64 *	0.5/4	1	<0.12/4	4 *	<0.015	<0.012	<0.03	4 **
TO30E	*Rahnella aquatillis*	Tomato	0.5	1	<0.06/4	<0.25	<0.12/4	<0.06	<0.015	<0.012	<0.03	4 **
AG24E	*Serratia fonticola*	Water	4	0.5	<0.06/4	<0.25	<0.12/4	<0.06	<0.015	<0.012	<0.03	8 **

FOX, cefoxitin; FOT, cefotaxime; FC, cefotaxime/clavulanic acid; TAZ, ceftazidime; TC, ceftazidime/clavulanic acid; FEP, cefepime; ETP, ertapenem; IMI, imipenem; MERO, meropenem; TRM, temocillin. * Above the resistance breakpoint. ** Intermediate value between susceptible and resistant breakpoint.

## Supplementary Materials

The following are available online at https://www.mdpi.com/article/10.3390/foods10112609/s1, Table S1: Primers and conditions used for PCR amplification of *bla_TEM_*, *bla_SHV_*, and *bla_CTX-M_* genes.
