# Supplementary material for: Extended-Spectrum Beta-Lactamase-Producing Enterobacteriaceae in Fresh Produce
Source: Foods. 2021 Oct 28;10(11):2609. doi: 10.3390/foods10112609 (PMC8619215; doi:10.3390/foods10112609)
Supplement: Supplementary file 1 [file foods-10-02609-s001.zip › foods-1424760-supplementary.pdf]

Supplementary Table S1

Primers and conditions used for PCR amplification of *blaTEM*, *blaSHV*, and *blaCTX-M* genes

Primer name	Sequence (5’-3’)	Annealing temperature	Amplicon size in bp
TEM-164.SE	tcgccgcatacactattctcagaatga	58 ºC	445
TEM-165.AS	acgctcaccggctccagatttat		
bla-SHV.SE	tgcgttatattcgcctgtg		747
bla-SHV.AS	tgctttgttattcgggccaa		
CTX-M-U1	atgtgcagyaccagtaargtkatggc		593
CTX-M-U2	tgggtraartargtsaccagaaycagcgg		
